# Pyrimidinergic Receptor Activation Controls *Toxoplasma gondii* Infection in Macrophages

**DOI:** 10.1371/journal.pone.0133502

**Published:** 2015-07-20

**Authors:** Aline Cristina Abreu Moreira-Souza, Ygor Marinho, Gladys Correa, Giani França Santoro, Claudia Mara Lara Melo Coutinho, Rossiane Claudia Vommaro, Robson Coutinho-Silva

**Affiliations:** 1 Laboratório de Imunofisiologia, Instituto de Biofísica Carlos Chagas Filho, Universidade Federal do Rio de Janeiro, Rio de Janeiro, RJ, 21941–902, Brazil; 2 Laboratório de Inovações, Terapias, Ensino e Bioprodutos (LITEB), Instituto Oswaldo Cruz, FIOCRUZ, Rio de Janeiro, RJ, 21.040–900, Brazil; 3 Instituto de Biologia, Universidade Federal Fluminense, Niterói, RJ, 24020–140, Brazil; 4 Laboratório de Ultraestrutura Celular Hertha Meyer, Instituto de Biofísica Carlos Chagas Filho, Universidade Federal do Rio de Janeiro, Rio de Janeiro, RJ, 21941–902, Brazil; 5 Instituto Nacional de Ciência e Tecnologia para Pesquisa Translacional em Saúde e Ambiente na Região Amazônica (INPeTAm/UFRJ), Rio de Janeiro, RJ, 21941–902, Brazil; University at Buffalo, UNITED STATES

## Abstract

Infection by the protozoan parasite *Toxoplasma gondii* is highly prevalent worldwide and may have serious clinical manifestations in immunocompromised patients. *T*. *gondii* is an obligate intracellular parasite that infects almost any cell type in mammalian hosts, including immune cells. The immune cells express purinergic P2 receptors in their membrane – subdivided into P2Y and P2X subfamilies - whose activation is important for infection control. Here, we examined the effect of treatment with UTP and UDP in mouse peritoneal macrophages infected with *T*. *gondii* tachyzoites. Treatment with these nucleotides reduced parasitic load by 90%, but did not increase the levels of the inflammatory mediators NO and ROS, nor did it modulate host cell death by apoptosis or necrosis. On the other hand, UTP and UDP treatments induced early egress of tachyzoites from infected macrophages, in a Ca^2+^-dependent manner, as shown by scanning electron microscopy analysis, and videomicroscopy. In subsequent infections, prematurely egressed parasites had reduced infectivity, and could neither replicate nor inhibit the fusion of lysosomes to the parasitophorous vacuole. The use of selective agonists and antagonists of the receptor subtypes P2Y_2_ and P2Y_4_ and P2Y_6_ showed that premature parasite egress may be mediated by the activation of these receptor subtypes. Our results suggest that the activity of P2Y host cell receptors controls *T*. *gondii* infection in macrophages, highlighting the importance of pyrimidinergic signaling for innate immune system response against infection. Finally the P2Y receptors should be considered as new target for the development of drugs against *T*. *gondii* infection.

## Introduction

Toxoplasmosis affects approximately one third of the world population [1], and is caused by the protozoan parasite *Toxoplasma gondii*. The disease is commonly asymptomatic in immunocompetent individuals, but has serious clinical manifestations in immunocompromised patients, including encephalomyelitis and multiple organ failure, leading to death in most cases, if not treated. Also, congenital toxoplasmosis in newborns can cause hydrocephalus, microcephaly and various central nervous system disorders, as well as recurring chorioretinitis [2].

*Toxoplasma gondii* is an obligate intracellular parasite capable of infecting all nucleated cell types, in humans and other homothermous hosts [3]. Intracellular pathogens such *T*. *gondii*, *Mycobacterium tuberculosis*, *Listeria monocitogenesis*, *Chlamydia sp*., *and Leishmania spp*. have developed strategies against host defense mechanisms that often rely on the ‘subversion’ of host molecules and pathways [4] Resistance strategies of *T*. *gondii* include the inhibition of phagolysosomal fusion—which prevents parasitophorous vacuole acidification and the attack by lysosomal proteolytic enzymes—by active exclusion of vacuolar fusion proteins from the membrane of the vacuole [5,6]. Indeed, after active entry into host cells, *T*. *gondii* tachyzoites remain inside the parasitophorous vacuole and escape killing by immune system cells by inhibiting the fusion of acidic organelles from the endo/exocytic pathways with the vacuole [7].

*Toxoplasma gondii* also modulate the host cell’s production of reactive oxygen species (ROS), inflammatory mediators involved in the control of intracellular infections [5,6] *T*. *gondii* express antioxidant enzymes, including catalases and peroxidases, to protect themselves against ROS activity [6,8,5]. The antioxidant system of *T*. *gondii* is composed of several enzymes (including catalase and peroxiredoxine) which completely blocks ROS production by host cells [5].

While ROS production contributes to pathogen elimination by destroying parasite structures by oxidation, this effect is potentiated by the formation of NO [4]. NO is a major mediator of resistance to *T*. *gondii* infection and innate and adaptive responses to NO, produced primarily by IFN-ɣ-activated phagocytes, are vital to control tachyzoite replication and differentiation into cystic bradyzoites, and for chronic disease establishment [9].

Calcium signaling is also important for *T*. *gondii* infection, since the parasite requires Ca^2+^ mobilization for host cell invasion, establishment in the parasitophorous vacuoles, recruitment of host cell organelles, and also for egress from infected host cells, at the end of intracellular replication cycles [10]. Interference with calcium signaling in the parasite can prevent host cell invasion, and treatment of infected cells with Ca^2+^ ionophore induces egress of the parasite after a short period of infection, or even after many replicative cycles [11].

The P2 family of nucleotide receptors includes G protein-coupled (GPCR) pyrimidine receptors from the P2Y subfamily, found in the plasma membrane of different cell types, including human and mouse immune cells [12]. P2Y_2_, P2Y_4_, P2Y_6_ receptors are coupled to G proteins from the Gq-subclass and their activation induces Ca^2+^ release from intracellular compartments via the classic phospholipase C pathway [13,14]. These receptor subtypes are expressed and functionally active in murine macrophages [15]. While P2Y_2_ is activated by ATP and UTP, P2Y_4_ and P2Y_6_ are activated by UTP and UDP, respectively. Upon cell lysis, exocytosis, or mechanical stress induced by hypoxia, these nucleotides are released into the extracellular medium [16], where they activate receptors from the P2 family by binding to their extracellular domain.

The presence of extracellular pyrimidine nucleotide agonists of P2Y receptors is associated with cytokine secretion, cell migration to inflammation sites [17,18], and also to immune responses against bacterial infections, where P2Y-mediated induction of MCP-1 chemokine expression leads to the recruitment of macrophages and monocytes to the infection site [19]. Previously, we showed that, during infection of macrophages with *Leishmania amazonensis*, the activation of P2Y receptors decreases parasite load in infected macrophages, in a Ca^2+^-dependent manner [20]. Despite the importance of Ca^2+^ signaling for different aspects of *T*. *gondii* infection, the ability of P2 receptors from the P2Y subfamily to modulate infection by this parasite has not been examined to date.

Considering that pyrimidinergic signaling in immune system cells controls infection by intracellular parasites, we evaluated whether the activation of P2Y receptors by the pyrimidine nucleotides UTP and UDP was also capable of modulating *T*. *gondii* infection. Our results show that activation of P2Y_2_, P2Y_4_ and P2Y_6_ receptors attenuates *T*. *gondii* infection in murine macrophages, by inducing premature, Ca^2+^-dependent egress of tachyzoite from host cells.

## Materials and Methods

### Animals and Parasites

BALB/c, C57Bl/6, Swiss CF1 or Swiss Webster mice (female or male) were purchased from the Multidisciplinary Centre for Biological Research (CEMIB, UNICAMP, Campinas, SP, Brazil). Mice aged between 8 and 12 weeks were used in all experiments, and were maintained at 22°C in a 12-h light/dark cycle. This study was carried out in strict accordance with the recommendations in the Guide for the Care and Use of Laboratory Animals of the National Institutes of Health (USA). The protocol was approved by the Committee on the Ethics of Animal Experiments of the Federal University of Rio de Janeiro (Permit Numbers: 039, 154 and 205). All efforts were made to minimize suffering. All the mice were euthanized through CO_2_ exposition followed by cervical dislocation.

Tachyzoites from the RH strain were obtained from the peritoneal cavity of Swiss CF1 mice 48 h post-infection.

### Reagents

Adenosine-50-triphosphate (ATP), adenosine diphosphate salt (ADP), uridine triphosphate salt (UTP), uridine diphosphate salt (UDP), MRS 2579, MRS 2693 and 2-Thio-UTP were from Tocris Bioscience (Bristol, UK). 40-6-diamidino-2-phenylindole (DAPI), hydrogenperoxide (H_2_O_2_), N-acetyl cysteine (NAC) and acridine orange, penicillin, streptomycin, HEPES, paraformaldehyde, glutaraldehyde, osmium tetroxide and bovine serum albumin (BSA) were purchased from Sigma Aldrich (St. Louis, USA). 20,70-dichlorofluorescein diacetate (H_2_DCFDA) and dihydroethidium (DHE) were purchased from Calbiochem (USA). Lactate dehydrogenize commercial kit was purchased from DOLES (Brazil). Acetone and ethanol were from Merk (Darmstadt, Germany). Fetal bovine serum (FBS) was from Gibco/life technologies, USA. The anti-LAMP-1-PE monoclonal antibody (cat. no. 12-1071-82 mAb) conjugate was purchased from e-Bioscience (Brazil). The anti-SAG-1 polyclonal antibody (pAb) was kindly provided by Dr. J.C. Boothroyd (Stanford University, USA), and the goat anti-rabbit-Alexa Fluor 488 secondary antibody was from Life Technologies (USA).

### Peritoneal Macrophages

Peritoneal exudate cells from BALB/c, C57Bl/6 or Swiss Webster mice were obtained by washing the peritoneal cavity with 8 ml of fresh DMEM. Peritoneal cells were counted in a hemocytometer and plated in 24-well plates containing 13-mm round coverslips or in cell culture flasks, and allowed to adhere in for 1 h, at 37°C in a humidified atmosphere with 5% CO_2_. Then, non-adherent cells were removed by washing with PBS and the medium was replaced. Macrophage cultures were maintained in fresh DMEM containing 10% fetal bovine serum (Gibco/life technologies, USA), 100 U/ml penicillin and 100 mg/ml streptomycin, and 10 mM HEPES, at 37°C, in a humidified atmosphere with 5% CO_2_.

### Infection and nucleotide treatments

*T*. *gondii* tachyzoites harvested from the peritoneal cavity of infected Swiss CF1 mice in PBS solution were centrifuged at 1000g for 10 min, resuspended in DMEM medium and allowed to interact with macrophages for 2 h, at a 3:1 or 5:1 ratio of tachyzoites to host cells. Then, extracellular parasites were removed by washes with PBS, and cells were incubated in medium containing 100 μM ATP, ADP, UTP or UDP, for 30 min. In some experiments, pre-treatment with 10uM of U73122, a phospholipase C inhibitor, were performed, 30 minutes previous to UTP treatment. After this period, the cells were washed and fixed, or maintained at 37°C in a humidified atmosphere with 5% CO_2_ for an additional 16 h. Infected cells were fixed in 4% paraformaldehyde and stained with Panótico Rapido” kit, (Laborclin, Brazil) following the manufacturer’s instructions. A minimum of 300 cells/sample were analyzed by light microscopy and the parasite load. Percentage of infected cells was determined using the formula: (iC x 100)/totalC, where iC is number of infected cells and totalC is the total number of cells. The infection index, which represents the number of parasites per infected cell, was determined using the formula: (% of infected cell X IntP)/totalC, where IntP is number of intracellular parasites.

For re-infection experiments, parasites that egressed prematurely from an initial round of macrophage infection were washed twice in sterile PBS and centrifuged at 1000g for 10 minutes, and then quantified in a hemocytometer. Egressed parasites were allowed to interact with fresh monolayers of peritoneal macrophages for 2 hours, and examined immediately or after 24 hours, by light microscopy (as described above). As a control for re-infection experiments, tachyzoites were allowed to interact with macrophages for 2 hours, and the parasites that had not entered macrophages during this period were collected and allowed to interact with fresh macrophage cultures, in identical conditions as those used for egressed parasites.

In some re-infection experiments, parasites that egressed prematurely from an initial round of macrophage infection were allowed to interact for 2 hours with fibroblasts, or with peritoneal macrophages that had been treated with 5 μM cytochalasin D for 30 minutes, and then examined by light microscopy (as describe above).

As a positive control for the experiments testing egressed parasite viability and infectivity, macrophages were infected with tachyzoites removed directly from the mouse peritoneal cavity (i.e., ‘fresh’ parasites).

### Imunofluorescence microscopy

Macrophages infected with *T*. *gondii* were fixed in 4% paraformaldehyde in PBS for at least 1 h, at room temperature. After washing with PBS, cells were permeabilized with 0.1% Saponin for 30 min at room temperature and with 100% acetone for 10 min at -20°C. Samples were blocked in 3% BSA/PBS blocker buffer for 40 min. Infected cells were then incubated 1 h with anti-SAG-1 (1:1000) and anti-LAMP-1-PE (0.2 mg/10^6^ cells) antibodies in blocker buffer. Then, samples were washed in 1% BSA/PBS for 15 min 3 times, incubated 30 min with blocker buffer, and labeled with goat anti-rabbit-Alexa Fluor 488 secondary antibodies (1:1000, for 1 h, at room temperature). Coverslips were washed gently with distillated water, mounted onto slides using Vectashield (Vector Labs. USA), and samples were analyzed in an Axiovert 200 microscope with an ApoTome fluorescence module (Carl Zeiss, Germany).

To evaluate phagolysosomal fusion during *T*. *gondi* infection, peritoneal macrophages were infected for 2 h as described above, incubated for 30 minutes with 100 nM of Lysotracker Red (Life Technologies, USA), and then fixed in 4% paraformaldehyde in PBS for 30 minutes. Coverslips were washed gently in distillated water, mounted onto microscope slides using Vectashield, and observed in an Axiovert 200 microscope with an ApoTome fluorescence module (Carl Zeiss, West Germany).

### Reactive oxygen species (ROS) assay

Macrophages that had been infected or not with *T*. *gondii* tachyzoites for 2 h were incubated with 10 mM N-acetyl cysteine (NAC) for 15 min at 37°C, followed by incubation with 20 μM of DHE for 30 minutes. Afterwards, cells were treated with 100 μM of UTP, UDP, ADP, ATP, or with 1mM of ATP as a positive control, and samples were analyzed for 30 min (at 5 min intervals) in a SpectraMax M2 spectrophotometer (Molecular Devices), at 37°C, excitation at 510 nm and emission at 595 nm.

### Nitric Oxide (NO) assay

To measure NO levels, the supernatant of infected and non-infected macrophages was collected after 4h of incubation with nucleotides. as described above (session 2.4), and nitrate levels were quantified using the Griess colorimetric method (for indirect NO quantification). Samples were analyzed in a SpectraMax M2 spectrophotometer (Molecular Devices), absorbance at 570 nm.

### Apoptosis assay

Peritoneal macrophages infected or not, as described in Section 2.4. were cultured for a further 10 h at 37°C (in 4% CO_2_) after treatment with different nucleotides. Then, cells were suspended with 200 μL of cellular cycle buffer (PBS containing 50 μg/mL ethidium bromide, 0.01g sodium citrate and 0.14% Triton X-100), and kept on ice for 15 minutes. As a positive control for apoptosis induction, cells were treated with 5 μM staurosporin for 24 h. Samples were analyzed (10,000 events/sample) using a FACSCalibur cytometer (BD, Germany), and flow cytometry data were analyzed using the WinMDI software (The Scripps Research Institute, La Jolla, USA). Cells with hypo-diploid amounts of DNA were considered apoptotic.

### Necrosis assay

Peritoneal macrophages infected or not, as described in Section 2.4 were cultured for a further 10 h at 37°C (in 4% CO_2_) after treatment with different nucleotides, and then 50 μL of supernatants were transferred to clear flat-bottom 96 well plates. Lactate dehydrogenase (LDH) activity was detected using a commercial kit (DOLES, Brazil), and absorbance was measured in a spectrophotometer, at 490nm. As a positive control for necrosis (regarded as 100% enzyme released), 1% Triton X-100 was added to cultures for a final concentration of 0.1%, 30 min before supernatant collection.

### Video Microscopy

Macrophages were cultured in 35-mm plates and infected with tachyzoites for 2 h. Infected cells were imaged by phase contrast (63x magnification, and 1.4 NA objective lens) in a CO_2_- and temperature-controlled chamber (5% of CO_2_ and 37°C) of a Zeiss Laser TIRF-2 microscope. Cells were observed for two minutes prior to the addition of 100 μM UTP to the culture medium, and then imaged for 10 min, at 12 frames/min. The sequence of images was optimizes by processing using the Fiji video editor.

### Transmission Electron Microscopy (TEM)

Macrophages were cultured in 6-well plates and infected with tachyzoites for 2 h. Then, cells were kept untreated or were treated with 100 μM UTP or UDP for 30 or 15 min, at 37°C. Sodium cacodylate buffer (0.1M, pH 7.4) was used for fixation, post-fixation and washes. Samples were fixed in 2.5% glutaraldehyde for at least 1 h at room temperature, washed and post-fixed in 1% osmium tetroxide/1.25% potassium ferrocyanide/5 mM CaCl_2_, for 50 min at room temperature. Then, cells were washed, dehydrated in series of acetone solutions and embedded in PolyBed resin (Polyscience Inc., Warrington, USA). Ultrathin sections were stained with uranyl acetate and lead citrate and observed in a Zeiss 900 transmission electron microscope (Carl Zeiss, Germany).

### Scanning Electron Microscopy (SEM)

Macrophages were cultured on coverslips in 12-well plates and infected with tachyzoites for 2 h. Infected cells remained untreated or were treated with 100 μM UTP or UDP for 15 min, at 37°C. Then, cells were fixed and post-fixed as described above (section 2.10), dehydrated in a series of ethanol solutions, critical point dried in an Bal-Tec CPD 030 (Balzers, Lichtenstein), mounted on metal supports and sputter coated with gold (Balzers sputter Union) for 1 minute. Alternatively, samples were extracted with 0.1% Triton-X-100 for 2 min [21] before fixation, or ‘dry-cleaved’ with carbon adhesive tape [22] prior to gold coating, to expose internal structures. Samples were observed in a Quanta 250 (FEI, USA) scanning electron microscope.

### Statistical Analyzes

All data were analyzed using unpaired Student’s t-tests, and p < 0.05 was considered statistically significant.

## Results

### Treatment with the P2Y agonist nucleotides reduces *T*. *gondii* infection in peritoneal macrophages

**Fig 1:**
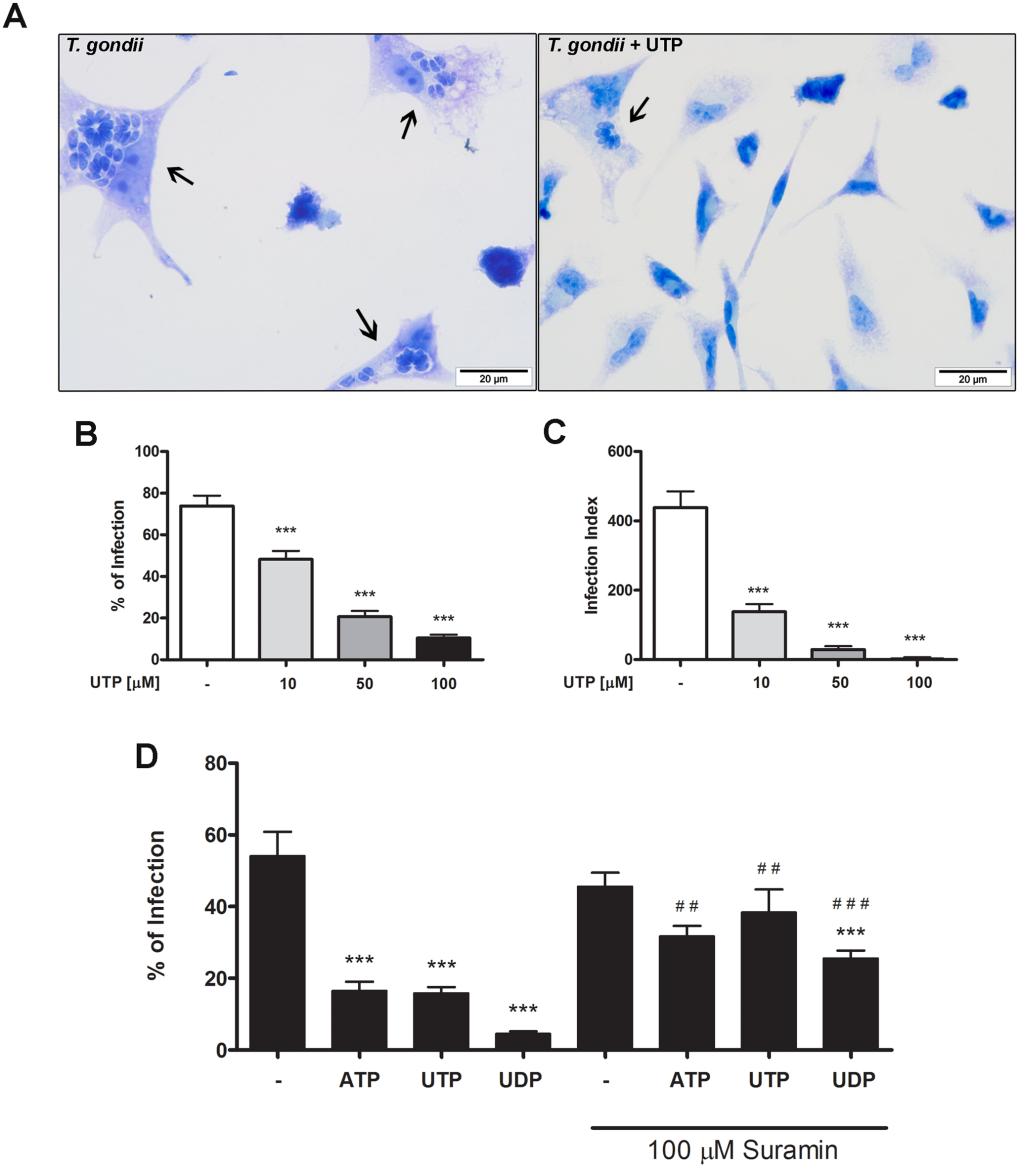
Treatment with the P2Y agonist nucleotides reduces *T*. *gondii* infection in peritoneal macrophages. Mouse peritoneal macrophages were infected with *T*. *gondii* tachyzoites for 2h and then treated with nucleotides for 30 minutes. (A) Infected cells stained with panotic, showing that the parasite load was reduced after 18h of infection. Black arrows indicate parasitophorus vacuoles containing *T*. *gondii* tachyzoites. (B-C) Treatment with UTP reduced the percentage of infected cells (B) and the number of parasites per host cell (infection index; (C), in a dose-dependent manner. Data represent standard error of mean (SEM) of five independent experiments (D) Nucleotide treatment reduced the % of infection, and this effect was reversed by pre-treatment with 100 μM of the P2 antagonist suramin (for 30 minutes before (100 μM) nucleotide treatment). Data represent mean and standard error of mean (SEM) of three independent experiments; * significantly different relative to untreated; #, significantly different relative to the corresponding nucleotide-treated group not pre-incubated with suramin. *,^#^ p < 0.05; * *, ^# #^ p < 0.001; * * *, ^# # #^ p < 0.0001.

To evaluate the role of P2Y receptors in the infection of macrophages by *T*. *gondii*, peritoneal macrophages from BALB/c mice were infected with tachyzoites at a ratio of 5:1 parasites per host cell, and then treated with increasing concentrations of the P2Y agonist UTP. In cells observed 18 h post-infection, treatment with UTP significantly reduced both the number of infected macrophages and the parasite load, in a dose-dependent manner (Fig 1A, 1B and 1C). Although most experiments were performed using macrophages from BALB/c mice, UTP treatment also protected macrophages from other lineages (C57BL/6 and Swiss webster) against *T*. *gondii* infection (S1 Fig), although the effect was more pronounced in BALB/c macrophages. Therefore, macrophages from BALB/c mice were used in all subsequent assays. Infected macrophages treated with UTP appeared better preserved and had a reduced number of parasites compared with untreated cells (Fig 1A). In addition, an increased proportion of cells remained attached in UTP-treated samples, compared with untreated ones (Fig 1A). This phenomenon was also observed in peritoneal macrophages of the C57BL/6 and Swiss Webster mouse strains (data not shown).

### The P2 receptor antagonist suramin reverses *T*. *gondii* infection reduction by P2Y nucleotide agonists

To evaluate the role of different members of the P2Y receptor family in the host response during infection by *T*. *gondii*, peritoneal macrophages infected with tachyzoites at a 5:1 ratio of tachyzoites to host cells were treated for 30 minutes with 100 μM of ATP (P2Y_2_ activator), UTP (P2Y_2_ and P2Y_4_ activator) or UDP (P2Y_6_ activator). Treatment with any of these P2Y agonists resulted in a reduction of parasite load in 70, 60 and 90% respectively after 18 hours of infection (Fig 1D). This effect was partially (for UDP) or totally (for ATP and UTP) reversed by pre-treatment with the P2 antagonist of suramin (100 μM, for 30 minutes), before treatment with nucleotides (Fig 1D).

### P2Y nucleotide agonists do not alter the production of nitric oxide (NO) and reactive oxygen species (ROS) by infected macrophages

**Fig 2:**
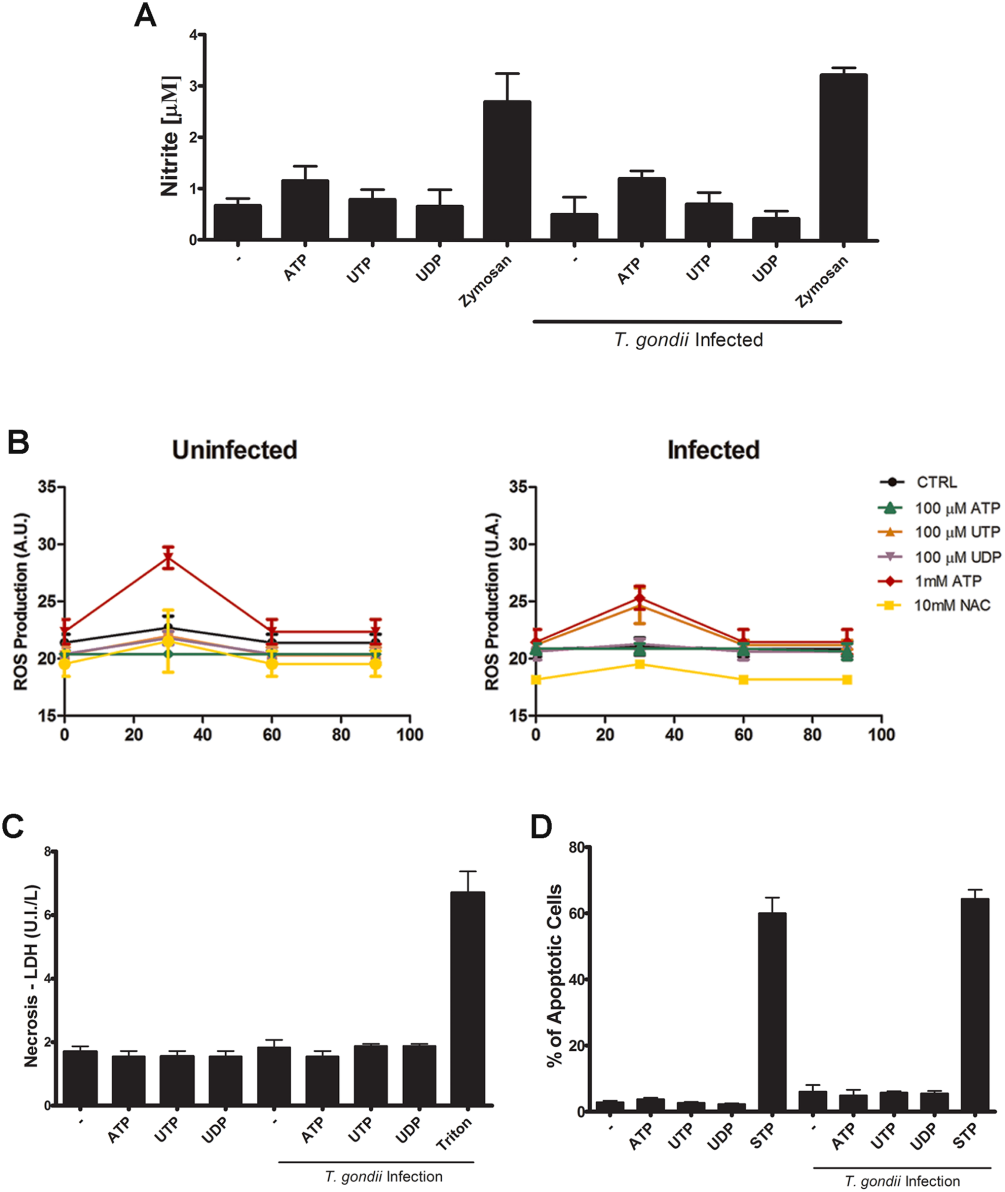
Effect of treatment with nucleotide agonists of P2Y receptors in the production of nitric oxide (NO), reactive oxygen species (ROS) and cell death by *Toxoplasma gondii*-infected macrophages. Mouse peritoneal macrophages were kept uninfected or were infected with *T*. *gondii* tachyzoites (at a 5:1 ratio of tachyzoites to host cells) for 2 h, and then treated with 100 μM of ATP, UTP or UDP during 30–90 min. Then, cell supernatants were analyzed for the levels of the inflammatory mediator NO 30 min later (A), and cells were analyzed for ROS production (B), using the Griess reagent method (indirect NO quantification via nitrite level measurements) and dihydroethidium (DHE) fluorescence, respectively. Neither nucleotide treatment nor infection resulted in statistically significant changes in NO levels (A). As expected, 1mM ATP increased ROS production by macrophages, although this effect was significantly less pronounced in infected cells (B). In contrast, other nucleotide treatments did not alter ROS production by uninfected or *T*. *gondii*-infected macrophages (B). Necrotic cell death was analyzed by measuring lactate dehydrogenase (LDH) activity in culture supernatants harvested 4h after nucleotide treatment (C). Treatment with 0.1% Triton X-100 was used as a positive control for necrosis. Nucleotide treatment did no induce necrosis in uninfected or *T*. *gondii*-infected cells. Apoptotic cell death was analyzed by flow cytometry using ethidium bromide, to identify cells with < 2C DNA content (D). As a positive control for apoptosis induction, cells were treated with 5 μM staurosporine (STP) for 24 h before analyze. Treatment with nucleotides for 12 h did not induced apoptosis in uninfected or *T*. *gondii*-infected macrophages. Data represent mean and SEM of three independent experiments.

To investigate the mechanism of nucleotide-mediated decrease in *T*. *gondii* infection burden in macrophages, we analyzed NO and ROS production by infected cells after treatment with 100 μM ATP, UTP or UDP (Fig 2). NO production was evaluated in cell culture supernatants collected 4 h post-infection, and treatment with 250 mg/mL zymosan was used as positive control. Nucleotide treatment did not induce NO production by uninfected or *T*. *gondii*-infected peritoneal macrophages (Fig 2A). These results suggest that the reduction in *T*. *gondii* infection burden induced by P2Y activation is not mediated by NO (Fig 2A).

The kinetics of ROS production by in uninfected or *T*. *gondii*-infected peritoneal macrophages (2h post-infection) was evaluated during treatment with 100 μM ATP, UTP or UDP (for 90 min, at 5-min intervals) (Fig 2B). Treatment with 100 μM ATP, UTP or UDP did not induce ROS production in infected or uninfected cells. In contrast, positive control treatment with 1mM of ATP increased ROS production in uninfected cells, and this effect was attenuated in infected cells (Fig 2B), and treatment with N-acetyl cysteine (NAC) was used as negative control.

### P2Y nucleotide agonists do not induce cell death in *T*. *gondii*-infected macrophages

To investigate if P2Y receptor activation is involved in cell death or protection of parasite induced cell lysis, we analysed cell death by necrosis in infected and uninfected cultures treated with 100 μM ATP, UTP or UDP for 30 min, and then incubated at 37°C further 10h. Measurements of lactate dehydrogenase (LDH) activity—a surrogate marker for necrosis—in culture supernatants indicated that necrosis levels did not increase as a result of infection, and that treatment with nucleotide agonists of P2Y receptors did not alter necrosis levels in uninfected or *T*. *gondii*-infected macrophage cultures (Fig 2C).

We also checked if treatment with P2Y receptor agonists induced apoptosis in *T*. *gondii*-infected macrophages. In peritoneal macrophage cultures infected or not with *T*. *gondii* for 2h and treated with P2Y receptors agonists the number of apoptotic cells (i.e., cells with hypo-diploid amounts of DNA, in flow cytometry analysis using ethidium bromide) remained as low as that observed in uninfected cultures, and nucleotide treatment did not induce apoptosis in uninfected or infected cells (Fig 2D). In contrast, treatment with staurosporine led to potent apoptosis induction (positive control).

### P2Y receptor agonists induce premature egress of tachyzoites from host cells

**Fig 3:**
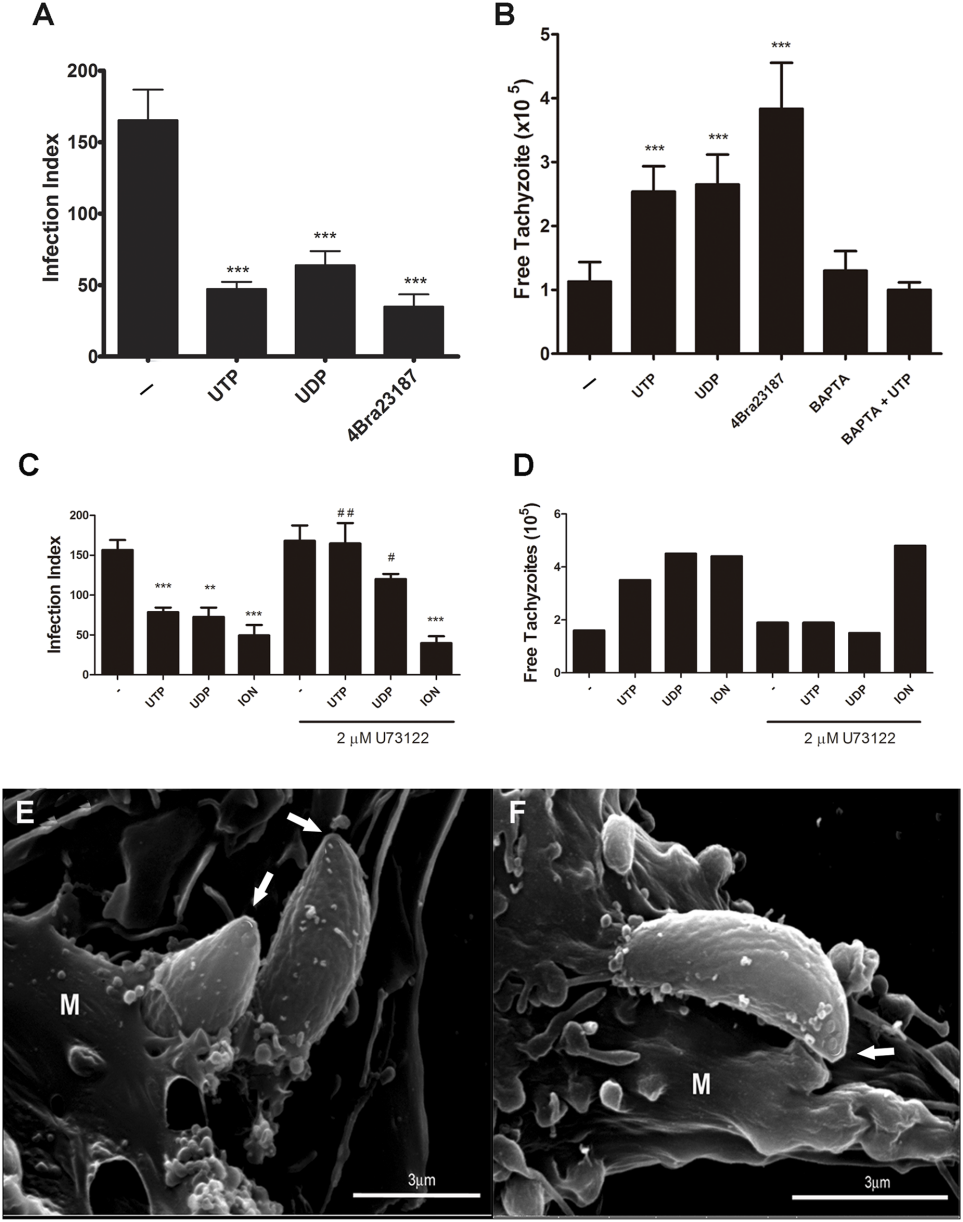
P2Y receptor agonists induce calcium-dependent premature egress of tachyzoites from host cells. Macrophages infected with *T*. *gondii* tachyzoites at a ratio of 5:1 parasites per host cell were treated for 30 minutes with 100 μM UTP or UDP, or for 15 minutes with 5 μM of the Ca^2+^ ionophore 4Bra23187. Also, some samples were pre-incubated with 2 mM of the calcium chelator BAPTA-AM or with 2 μM of the phospholipase C inhibitor U73122 for 30 minutes, before UTP treatment. Cells were processed for light microscopy analysis of the infection index (A and C), and the number of free tachyzoites in culture supernatants was determined using a hemocytometer (B and D). Infected macrophages (M) were treated with 100 μM of UTP or UDP for 15 minutes and then processed for scanning electronic microscopy. The images show parasites activelly egressing from host cells (E and F), with extruded conoids (arrows). (A, B, C) Data represent mean and SEM of ten (A), seven (B) and three (C) independent experiments. (D) Data are representative of two independent experiments. ** p<0.001 and *** p < 0.0001 relative to untreated. ## p<0.01 and ### p<0.0001 relative to treated in presence of phospholipase C inhibitor.

Since the reduction in *T*. *gondii* macrophage infection induced by P2Y agonist treatment was not due to increased NO or ROS production, or cell death induction, we hypothesized that tachyzoites might be egressing prematurely from infected cells upon nucleotide treatment. To test this hypothesis, we determined the infection index in cultures (2 h post-infection) fixed immediately after treatment with 100 *μ*M UTP or UDP for 30 minutes (Fig 3A), and also quantified the number of parasites observed in the culture medium after treatment (Fig 3B). We observed that both UTP and UDP treatment reduced the number of parasite inside of cells with an overall reduction in parasite burden of 38% and 33%, respectively (Fig 3A). Ca^2+^ influx into host cells induces *T*. *gondii* egress even after a short period of infection [11]. Thus, treatment with the Ca^2+^ ionophore 4BrA23187 (for 15 min) was used as a positive control for early tachyzoite egress from host cells. Similarly to that observed after treatment with both UTP and UDP treatments 4BrA23187, reduced the number of infected macrophages and the infection index, (Fig 3A).

Although infection index reduction immediately after P2Y agonist treatment suggested that tachyzoites egressed prematurely from host cells, it was important to verify whether tachyzoites were indeed released into the medium after treatment. For these experiments, we also treated cells with the Ca^2+^ chelator BAPTA-AM (for 30 min) prior to treatment with nucleotides or with 4BrA23187, to examine the possibility that early parasite egress after nucleotide treatment was dependent on a Ca^2+^ influx. Culture supernatants of infected cells treated with UTP, UDP or 4BrA23187 contained a larger number of parasites than untreated cell supernatant. Importantly, this effect was reduced by pre-treatment of cells with BAPTA-AM before UTP treatment (Fig 3B). These results suggest that the activation of P2Y receptors on the surface of *T*. *gondii*-infected macrophages induced early parasite egress from host cells, in a Ca^2+^-dependent manner.

To examine the mechanisms of nucleotide-mediated parasite egress, we performed UTP treatments in the presence of U73122, a phospholipase C inhibitor expected block the P2Y intracellular signaling cascade. We found that the pre-treatment with U73122 completely abolished the UTP-mediated parasite egress, but had no effect on the response to Ca^2+^ ionophore treatment (Fig 3C and 3D). These results confirm that the UTP-response depends on P2Y receptor signaling, via intracellular calcium mobilization.

Videomicroscopy analysis showed tachyzoites egressing from infected macrophages right after nucleotide treatment. This phenomenon was not seen in untreated cultures (S1 Video).

Parasite egress was also evident by Scanning Electron Microscopy analysis of infected cells treated with nucleotides (Fig 3E). Extrusion of the conoid structure is an important event during active tachyzoite exit from (and also entry into) host cells [10] [23,24]. SEM images of parasites egressing shortly after treatment with UTP or UDP (Fig 3C) show an extruded conoid in the extracellular milieu (suggestive of active egress), while the posterior end of the parasite remains inside the host cell.

**Fig 4:**
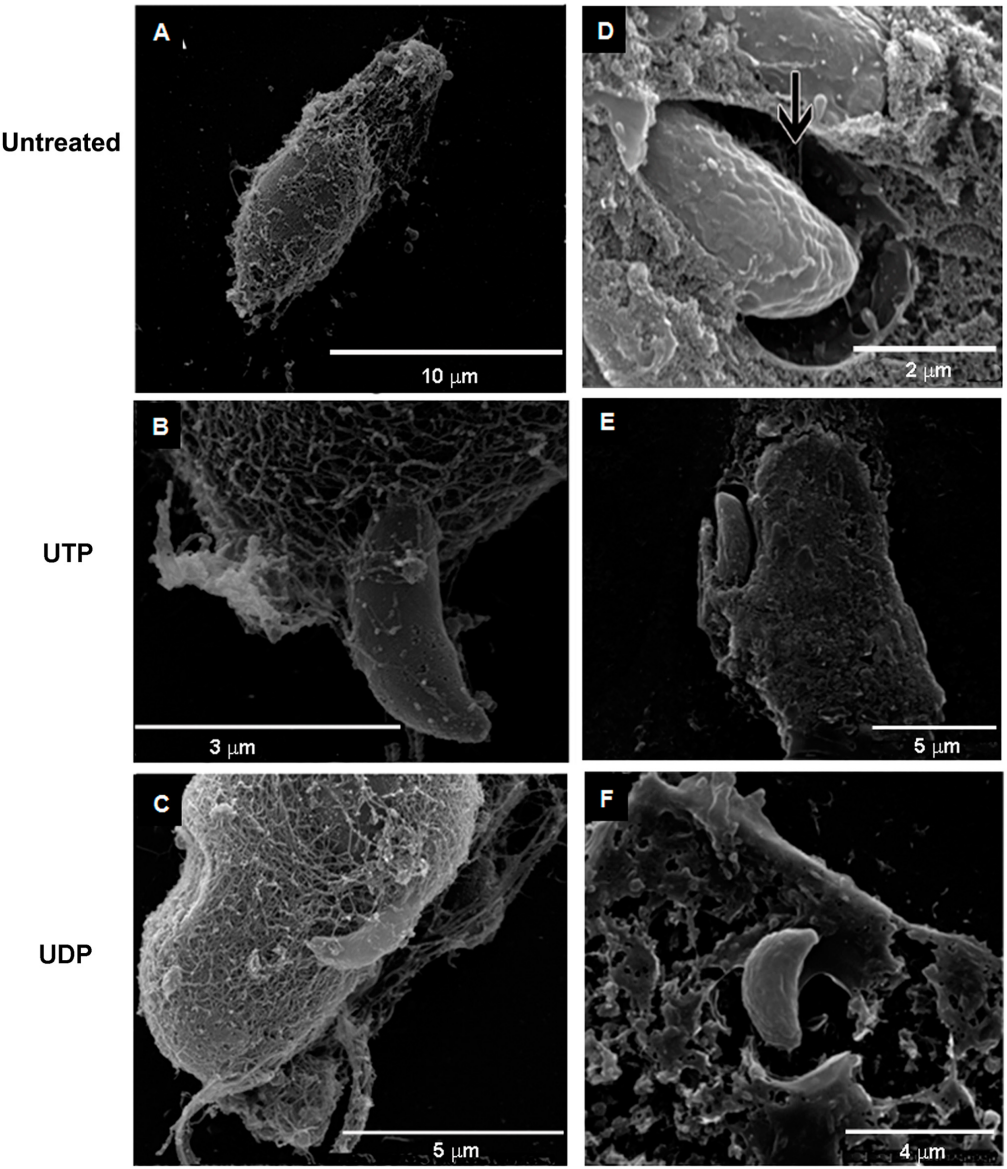
Scanning Electron Microscopy (SEM) of *Toxoplasma gondii*-infected macrophages treated with UTP or UDP. Mouse peritoneal infected-macrophages treated with UTP or UTP for 15 minutes were incubated with 0.1% triton X-100 for 2 min before fixation for SEM (A-C), to remove the host cell plasma membrane, or dry-cleaved with adhesive tape (D-F), to expose the cytoplasm containing parasites. (A) the micrograph shows no visible parasite out of parasitophorus vacuoles. Figs (B) and (C) show parasites interacting with cytoplasmic structures. (D) the micrograph shows a parasite inside a parasitophorous vacuole, and interacting with the intravacuolar network (arrow), as expected during normal infection. Figs (E) and (F) shows egressing parasite from UTP- and UDP-treated cells, respectively, with extruded conoid structure, typical of parasites in active egress.

To visualize the internal macrophage structures at the site of egress, the plasma membrane of *T*. *gondii*-infected cells was removed by treatment with a 0.1% Triton X-100 for 2 minutes prior to fixation for SEM. In control cells, parasites remained inside the parasitophorous vacuoles (Fig 4A), whereas in treated cells, parasites appear to interact directly with cytoplasmic structures, presumably the host cytoskeleton (Fig 4B and 4C). To visualize parasites inside treated host cells, some SEM samples were dry-cleaved with adhesive tape prior to gold coating. In untreated cells, tachyzoites were inside parasitophorous vacuoles and appeared to interact with the ‘intravacuolar network’ (Fig 4D). In contrast, in macrophages treated with UTP and UDP, tachyzoites appeared to be outside parasitophorous vacuoles, interacting directly with the host cell’s cytoplasm (Fig 4E and 4F). These results suggest that treatment with nucleotides induced exit of *T*. *gondii* tachyzoites from host cell, soon after infection. In ultrathin TEM sections, infected cells treated with 100 μM of UTP for 30 minutes showed no parasitophorous vacuoles (data not shown) while untreated cells had parasitophorous vacuoles containing seemingly viable parasites, typically surrounded by macrophage organelles and lipid inclusions (data not shown).

### *T*. *gondii* tachyzoites that egress prematurely from nucleotide-treated macrophages have reduced infectivity

**Fig 5:**
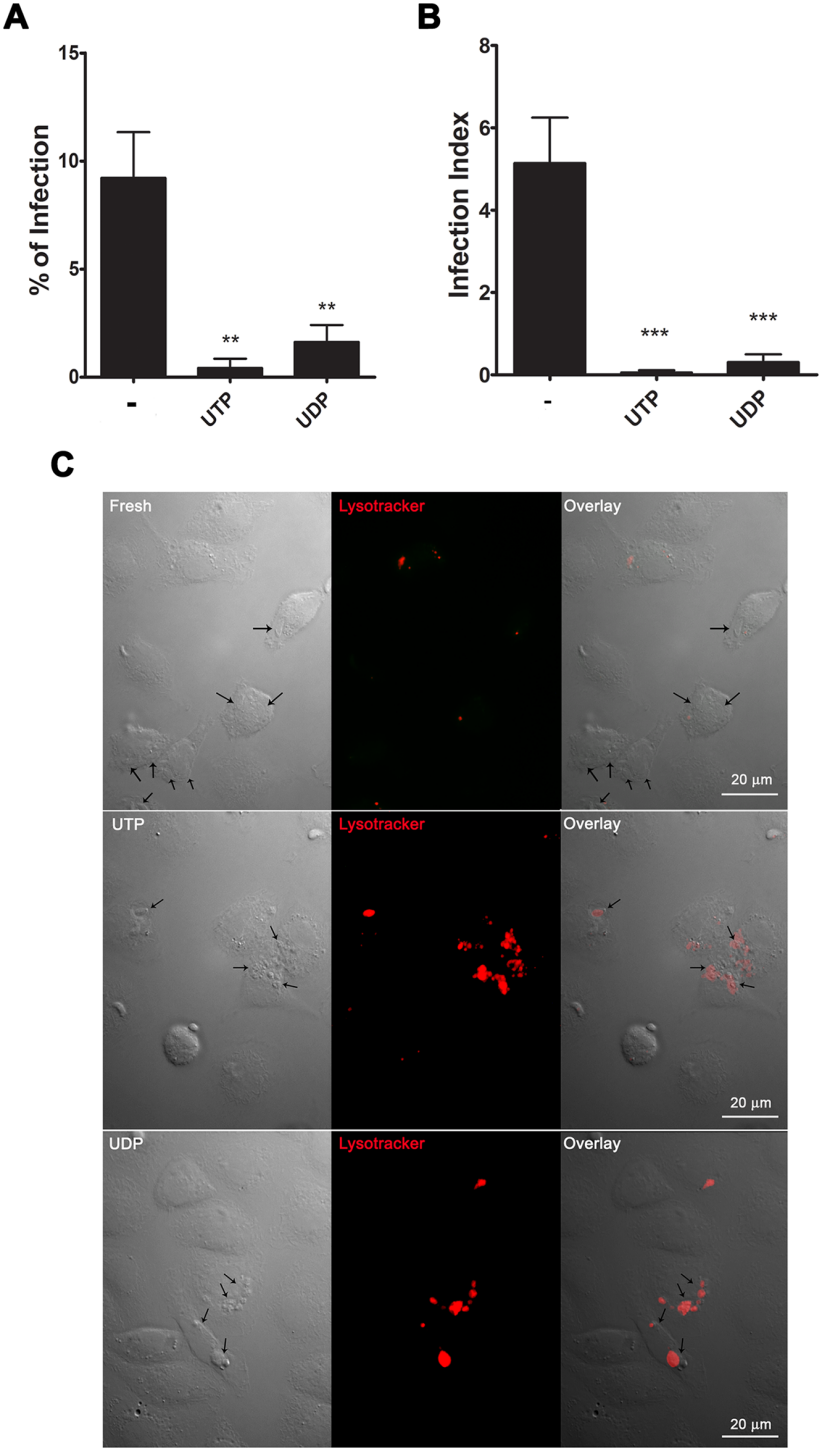
*T*. *gondii* tachyzoites that egress prematurely from nucleotide-treated macrophages have reduced infectivity. Mouse peritoneal macrophages infected with tachyzoites at a 5:1 ratio were treated with 100 μM UTP or UDP for 30 min. Immediately after nucleotide treatment, prematurely egressed parasites were recovered from culture supernatants and allowed to interact with cultures of freshly harvested peritoneal macrophages for 24 h. Cells were then processed for light microscopy analysis of the % of infected cells (A) and the infection index (B). Untreated control parasites represented those that had not invaded the untreated cells 2 h post-interaction. Cytoplasmic vacuole acidification was analyzed by fluorescence microscopy using the acidic compartment probe Lysotracker red (C). Arrows indicate parasites inside host cells. Phagolysosomal fusion inhibition (with lack of Lysotracker red staining) was observed in cells infected with control parasites obtained from infected mice (Fresh). In contrast, in cultures infected with parasites rescued from nucleotide-treated cells (UTP or UDP), tachyzoites were found inside acidic (Lysotracker red-positive) parasitophorous vacuoles, indicating that phagolysosomal fusion occurred during infection. (A, B) Data represent mean and SEM of three independent experiments. * p < 0.05; ** p < 0.001; *** p < 0.0001, relative to untreated controls.

By videomicroscopy, tachyzoites that had egressed from nucleotide-treated cells prematurely appeared slower or almost motionless when compared with parasites egressing from untreated cells (data not shown). To verify if parasites egressing from nucleotide-treated cells remained infective, we allowed these parasites to interact with freshly harvested peritoneal macrophages. Tachyzoites recovered from the culture supernatant of UTP- and UDP-treated and untreated macrophages were washed twice in DMEM to remove residual nucleotides from the first round of interaction with host cells. Then, rescue parasites were kept with freshly harvested macrophages for two hours. After 24h these cells were processed for light microscopy and the parasite load was quantified. Tachyzoites that had egressed prematurely from UTP- or UDP-treated macrophages had reduced ability to re-infect cells (< 2% of the cells), in opposition to control tachyzoites recovered from the culture supernatant of untreated macrophages, 11% (Fig 5A). Also, the infection index was reduced in cultures infected with prematurely egressed parasites, suggesting that these parasites could not proceed with a new replicative cycle after host cell invasion (Fig 5B).

We also examined if the prematurely egressed parasites that managed to invade freshly harvested macrophages retained the ability to inhibit phagolysosomal fusion, as observed during normal *T*. *gondii* infection [25,26]. As a positive control for phagolysosomal fusion inhibition, we used tachyzoites obtained directly from the peritoneal cavity. In cells infected with control tachyzoites, cytoplasmic vacuole acidification (as identified by fluorescence using the acidic compartment probe Lysotracker red) was inhibited, and this effect was evident in the parasitophorous vacuole, since tachyzoites did not co-localize with Lysotracker red-labeled structures (Fig 5C). In contrast, tachyzoites that had emerged prematurely from a first round of infection as a result of nucleotide treatment were found within acidic Lysotracker red-labeled structures (Fig 5C), which suggested that these parasites were unable to inhibit phagolysosomal fusion.

**Fig 6:**
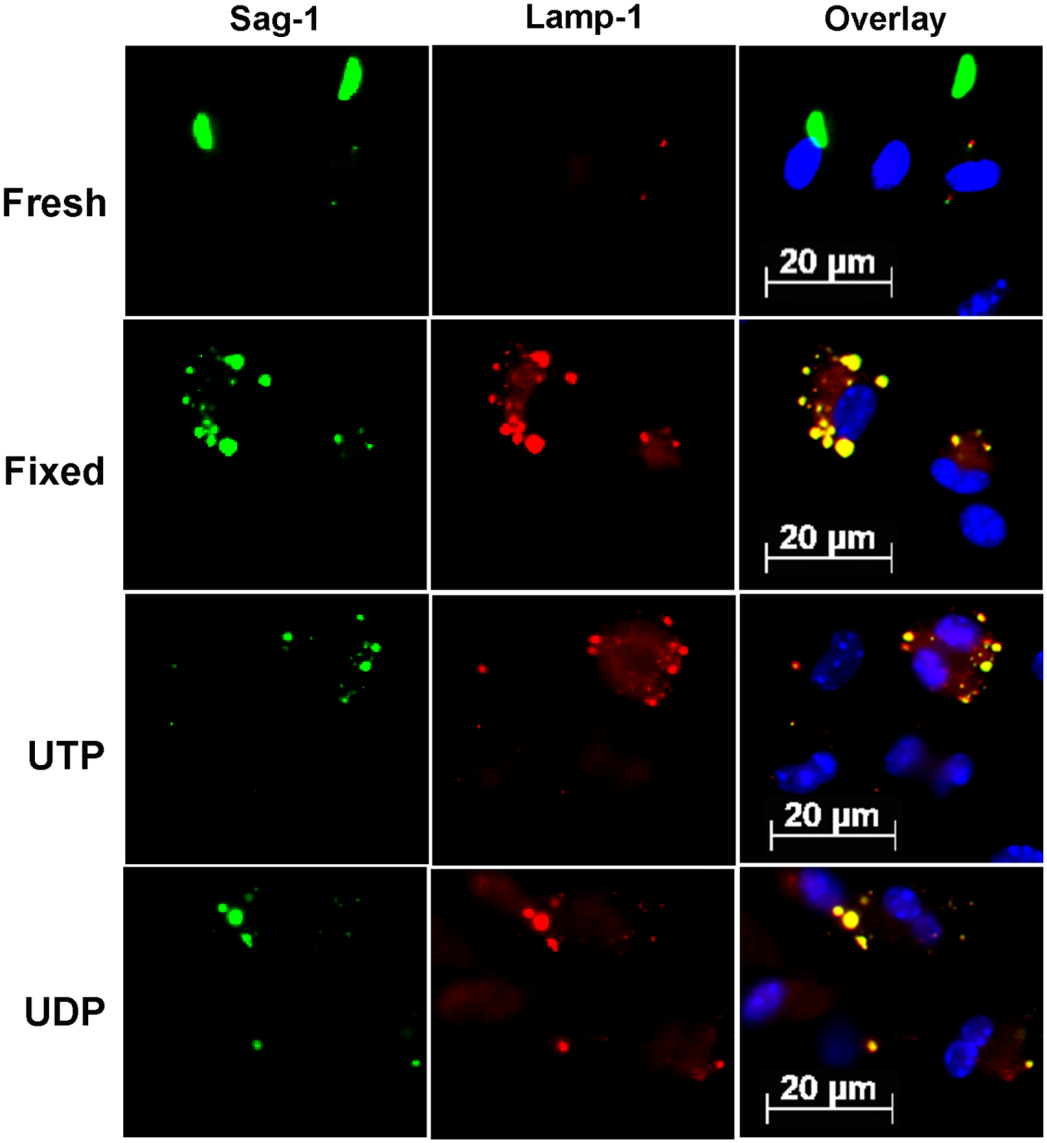
*Toxoplasma gondii* tachyzoites that egressed prematurely from nucleotide-treated cells could not inhibit phagolysosomal fusion in subsequent infections. Parasites that egressed prematurely from infected and nucleotide treated cells were recovered from culture supernatants and allowed to interact with freshly harvested macrophages for 2 h, and then processed for immunofluorescence to detect the tachyzoite surface protein SAG-1 (in green) and the lysosomal membrane protein LAMP-1 (in red). Phagolysosome fusion occurred in cells infected with prematurely egressed parasites (UTP and UDP samples), as evidenced by SAG-1 and LAMP-1 co-localization (yellow in the overlay). Phagolysosomal fusion also occurred in cells containing fixed tachyzoites (Fixed; positive control for fusion), but did not occur in cells infected with parasites freshly harvested from infected mice (Fresh; negative control for fusion).

To confirm that phagolysosomal fusion was allowed to proceed in cells infected with prematurely egressed tachyzoites, these cells were labeled with specific antibodies against the lysosomal marker LAMP-1 (lysosomal associated membrane protein-1) and the tachyzoite surface protein SAG-1 (Fig 6). In freshly harvested macrophages infected for 1h with tachyzoites that had egressed prematurely from UTP or UDP-treated cells, we observed co-localization of SAG-1 and LAMP-1 (Fig 6), suggesting that parasites were found within phagolysosomal structures. Co-localization was also observed in cells incubated with pre-fixed parasites (positive control for phagolysosomal fusion; (Fig 6), but was absent in cells infected with parasites freshly recovered from mice (negative control for phagolysosomal fusion).

**Fig 7:**
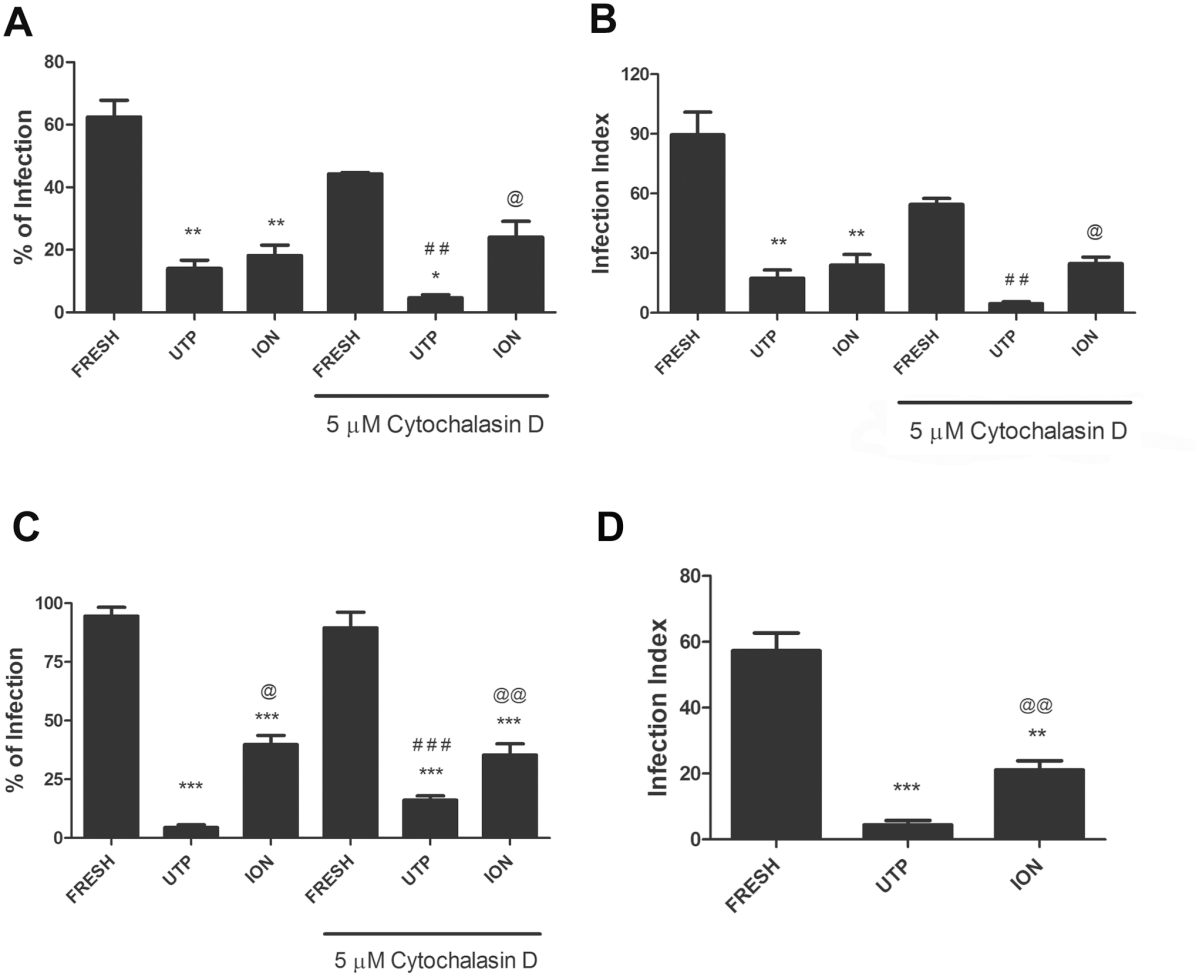
*Toxoplasma gondii* tachyzoites that egressed prematurely from nucleotide-treated cells are captured passively by host cells. Mouse peritoneal macrophages infected with tachyzoites at a 5:1 ratio were treated with 100 μM UTP (for 30 min) or 5 μM of the Ca^2+^ ionophore ionomycin (for 15 minutes). After nucleotide or ionophore treatment, prematurely egressed parasites were recovered from culture supernatants and allowed to interact with freshly harvested peritoneal macrophages, for 2 hours. Samples were examined either immediately (A and B) or 24 hours after treatment with cytochalasin D (C). The % of infected cells (A and C) and the infection index (B) were then estimated by light microscopy analysis. (D) Egressed parasites were allowed to interact with fibroblast cultures for 2 hours, and then the infection index was estimated by light microscopy analysis. * p < 0.05; ** p < 0.001; *** p < 0.0001, relative to samples “Fresh”. ## p < 0.001; ### p < 0.0001, relative to samples not treated with cytochalasin D. @ p<0.05; @@ p<0.001, for UTP vs Ca^2+^ ionophore treatments.

To verify if the egress of parasites induced by UTP was mechanistically related to that induced by Ca^2+^-ionophore, we performed parallel reinvasion experiments using egressed parasites from these two experimental conditions. We observed similar reinvasion rates (~70% less than that observed with fresh parasite) for both Ca^2+^ ionophore- and UTP-treated parasites (Fig 7A and 7B). However, the reinvasion of parasites that egressed as a result of UTP treatment was significantly reduced in macrophages pre-treated with cytochalasin D, an inhibitor of actin polymerization and, consequently, of phagocytosis (Fig 7A and 7B). In contrast, cytochalasin D treatment did not alter the reinvasion rate of parasites egressed by Ca^2+^ ionophore treatment (Fig 7A and 7B). In addition, parasites egressed by UTP treatment were capable of replicating inside macrophages 24 hours after infection of macrophages that had been pre-treated with cytochalasin D (Fig 7C).

To confirm if the active invasion was compromised we performed re-infection experiments in non-phagocytic HFF fibroblast cells line. We found that parasite egressed from UTP and Ca^2+^ ionophore treatment had reduced ability to actively invade the host cell. Therefore the reduction was more pronounced in egressed parasites from UTP-treatment (Fig 7D).

### Non-egressed parasites that remain inside nucleotide-treated macrophages do not proliferate

**Fig 8:**
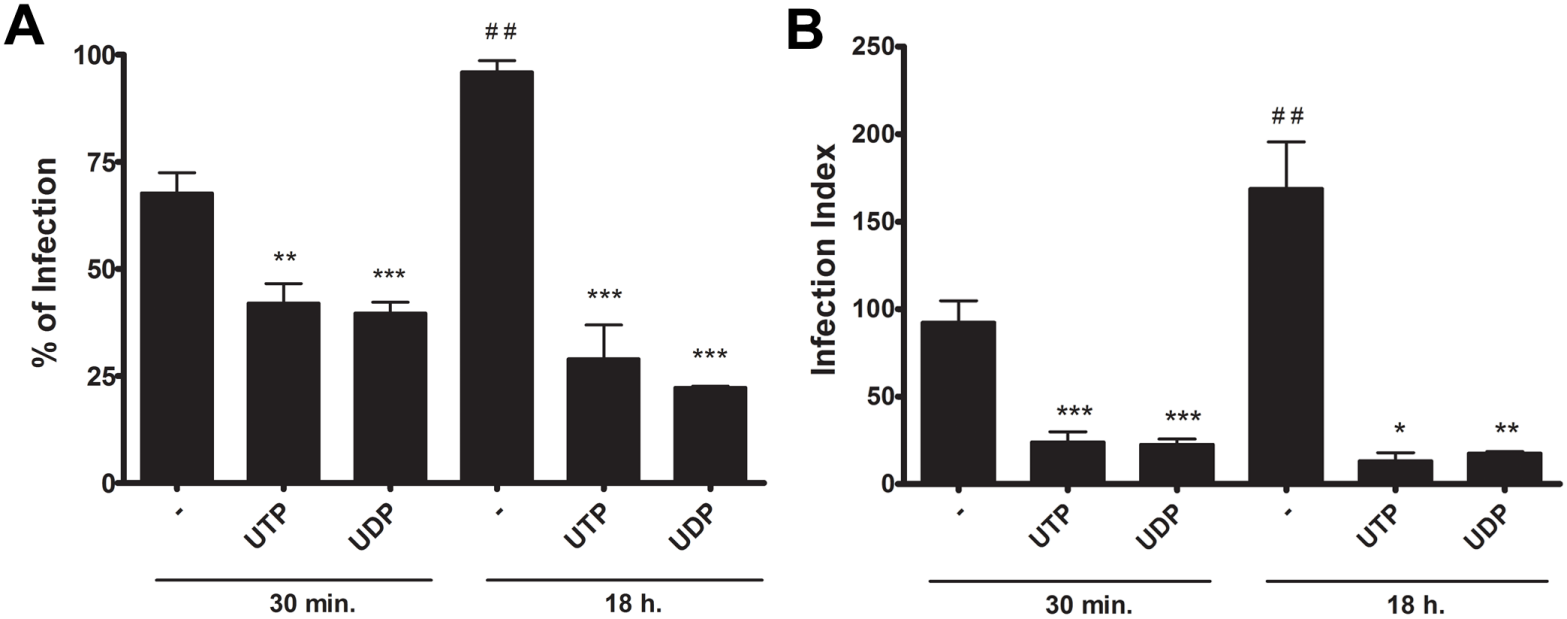
Non-egressed parasites that remain inside nucleotide-treated macrophages do not proliferate. Mouse peritoneal macrophages infected with tachyzoites at a ratio of 3:1 were treated with 100 μM UTP or UDP and then cultivated for 30 min or 18h prior to infection index determination, by light microscopy examination of panotic-stained cells. The figure shows the percentage of infected cells (A) and the number of parasites per host cell (infection index) (B); after 30 minutes of treatment, and after 18 h of infection. These results show the non-egressed parasite did not replicate after nucleotide treatment. Data represent mean and SEM of six independent experiments. *significantly different relative to untreated; #, significantly different relative to 30 min untreated * p < 0.05; **,^##^ p < 0.001; *** p < 0.0001.

After removal of egressed parasites from the supernatant of infected cultures treated with nucleotides, these cultures were maintained for 30 min or 18 h and then the infection index was estimated, to evaluate the fate of non-egressed tachyzoites (Fig 8). Our data suggest that the parasites that remained inside nucleotide-treated macrophages did not proliferate (Fig 8A and 8B). Thus, nucleotide treatment interferes with the parasite’s proliferative cycle, possibly through the activation of P2Y receptor signaling.

### P2Y_2_, P2Y_4_ and P2Y_6_ receptor subtypes are involved in the response to nucleotide treatment

P2Y receptors from the subtypes P2Y_2_, P2Y_4_ and P2Y_6_ are activated by UTP (P2Y_2_ and P2Y_4_) and UDP (P2Y_6_). Thus, to address the involvement of these receptor subtypes in early parasite egress after nucleotide treatment, and to evaluate the individual contribution of each subtype in early egress, we treated infected cells with selective agonists and antagonists of P2Y receptors and then determined the infection index.

**Fig 9:**
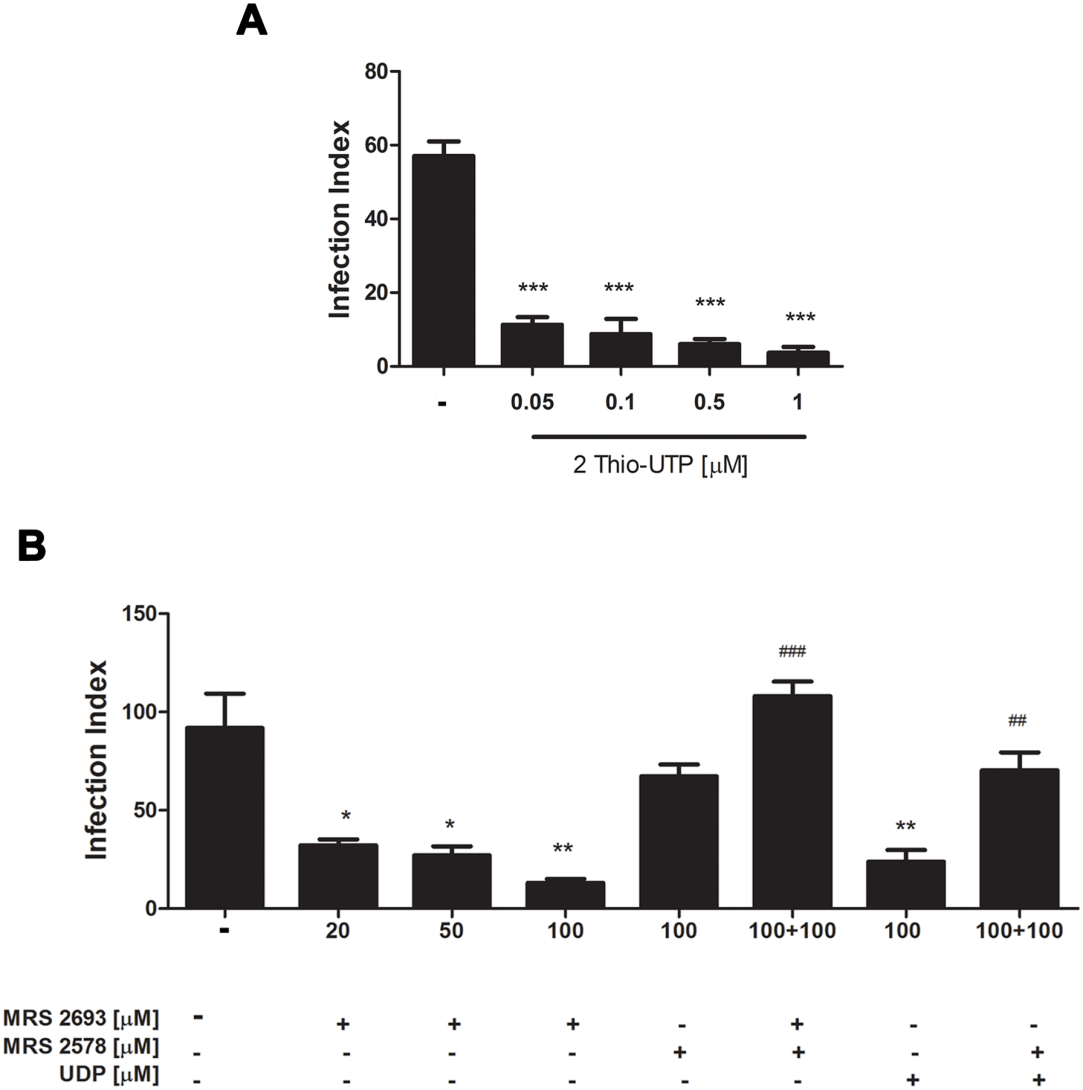
The activation of different P2Y receptor subtypes reduce *T*. *gondii* infection in peritoneal macrophages. Mouse peritoneal macrophages were infected with *T*. *gondii* tachyzoites at a ratio of 5:1 for 2 h and then treated with specific P2Y receptor agonists and antagonists for 30 min, prior to infection index determination. (A) Treatment 2 Thio-UTP at concentrations of 0.05 or 0.1 μM (to activate P2Y_4_) and 0.5 or 1 μM of 2 Thio-UTP (to activate P2Y_2_) led to similar reductions in the infection index relative to untreated controls. (B) The infection index was also reduced after treatment with MRS 2693, a specific P2Y_6_ agonist, and MRS 2693 effect was totally blocked by pre-treatment with the specific P2Y_6_ antagonist MRS 2578. The 20, 50, 100 represent on the x-axis concentration of the agonist/antagonist. * statistically significant relative to untreated. # statistically significant relative to reduction of infection index. Data represent mean and SEM of three independent experiments. * p < 0,05; **,^##^ p < 0.001; ***, ^###^ p < 0.0001.

The agonist 2Thio-UTP activates P2Y_2_ and P2Y_4_ at different concentrations [27]. Similar reductions in the infection index (82%) that were observed in UTP treatment were observed in infected macrophages treated with 2Thio-UTP at the concentrations of 0.05 and 0.1 μM, which trigger P2Y_2_ activation only, and at the concentrations of 0.5 and 1 μM, which activate both P2Y_2_ and P2Y_4_ (Fig 9A).

To define the contribution of P2Y_6_ receptors in the early nucleotide-induced *T*. *gondii* egress, we treated infected cells with the selective P2Y_6_ agonist MRS 2693. Treatment with MRS 2693 at concentrations of 20, 50 and 100 μM resulted in a dose-dependent decrease (of 30 to 56%) in the infection index (Fig 9B). This effect was completely reversed by treatment with the selective P2Y_6_ antagonist MSR 2578 before P2Y_6_ activation by MRS 2693 or UDP. In addition, we found that the UTP effect is not blocked by the selective antagonist of P2Y6 (data not shown). This finding reinforces the interpretation that UTP effects are truly mediated by UTP and are not a result of UDP generated by the action of NTPDases.

## Discussion

*Toxoplasma gondii* is an opportunistic parasite that causes severe diseases in immunocompromised patients, and no effective drug is available for the complete elimination of the parasite’s resistant intracellular cysts. P2Y receptors are widely expressed in immune cells and are involved in the response to inflammatory and parasitic diseases [12,28]. In a previous study, we showed that the activation of purinergic P2X7 receptors in macrophages leads to increased ROS production and elimination of intracellular *T*. *gondii* [29]. Here, we show that activation of receptors from the P2Y subfamily by the pyrimidine nucleotides UTP and UDP is also capable to control *T*. *gondii* infection in macrophages. However, UTP and UDP treatments did not induce ROS production in infected cells (Fig 2). Thus, ROS inflammatory mediators do not appear to contribute to the anti-*T*. *gondii* effect observed after UTP and UDP treatment, indicating that P2X and P2Y receptors are capable of modulating *T*. *gondii* infection via different mechanisms.

During macrophage infection by *Leishmania amazonensis*, the activation of P2Y receptors induced NO production by the phagocytes [20]. However in macrophages infected with *T*. *gondii*, activation of P2Y receptors did not result in increased NO production, suggesting that this microbicidal molecule is not involved in the anti-*T*. *gondii* effect observed after treatment of infected macrophages with P2Y receptor agonists.

Cell death by apoptosis participates in the immune response to infections by modulating *T*. *gondii* proliferation [25,26]. *T*. *gondii* infected macrophages have altered responses to programmed cell death stimuli [30,31,32], and D'Angelillo and collaborators suggested that *T*. *gondii* induces apoptosis in monocytes via autocrine TGF-β signaling [33]. Previously, we showed that P2 receptor activation by ATP treatment induces apoptosis in cells infected with *Mycobacterium sp*., *Chlamydia sp*. and *Leishmania* [34], and Marques-da-Silva and collaborators (2011) reported that P2Y receptor activation by UTP also induced apoptosis in macrophages infected with *Leishmania amazonensis* [20]. In the present study, we did not observe appreciable apoptosis or necrosis in UTP-treated macrophage cultures infected with *T*. *gondii* (Fig 2). Thus, infection reduction in these cells is not mediated by cell death modulation.

Overall, our data strongly suggest that UTP and UDP treatments attenuate *T*. *gondii* infection in peritoneal macrophages by inducing premature parasite egress from host cells. Ca^2+^ is vital for different aspects of *T*. *gondii* infection [10], and Ca^2+^ homeostasis is carefully controlled by the parasite, by manipulating Ca^2+^ storage in the endoplasmic reticulum, the mitochondrion and the acidocalcisomes [23,24]. Interference with calcium signaling in *T*. *gondii* prevents host cell invasion, and Ca^2+^ influx into host cells triggered by Ca^2+^ ionophore treatment induces artificial parasite egress [11]. The supernatant of *T*. *gondii*-infected cultures treated with UTP contained egressed parasites (Fig 3B), and SEM analysis showed parasites clearly egressing from macrophages as early as 15 minutes after nucleotide treatment (Fig 3C). Tachyzoites egressing prematurely from UTP- or UDP-treated cells displayed an extruded conoid similar to that observed in parasites egressing naturally at the end of lytic cycles [23,24], or artificially after Ca^2+^ ionophore treatment [11].

In line with the ability of P2Y receptors to modulate intracellular Ca^2+^ levels, we observed that the premature egress of tachyzoites from infected cells treated with UTP or UDP was dependent on Ca^2+^ (Fig 3). Premature egress was similar to that observed using Ca^2+^ ionophore, and was inhibited by treatment with the Ca^2+^ quelator BAPTA-AM or the phospholipase C inhibitor U73122.

As stated above, all major stages of the life cycle of the parasite are associated with the modulation of the host, which basically occurs by the secretion of the secretory organelles contents in the cytoplasm of the host or within the parasitophorous vacuole [10,24]. The secretion of microneme molecules and the trigger of the invasion machinery are associated to calcium influx [23,35]. Thus the activation of P2Y receptors and early egress might have compromised this mechanism impairing *T*. *gondii* invasion. However, the reinvasion data showed that most parasites that egress cells prematurely after UTP-treatment enter cells passively—likely by phagocytosis—during a subsequent encounter with host cells. Further analysis is now necessary to determine whether prematurely egressed parasites are no longer viable or have lost their ability to actively infect cells, but remain viable.

In addition, the mechanisms on how Ca^2+^ recruitment in either the host cell or the parasite are involved in inducing parasite egress [10,11] is not completely understood, but seems to be specifically due to the K^+^ efflux from host cells as shown in fibroblasts [36], and this mechanism seems to be independent of parasite motility and dependent on membrane tension [37]. This finding might help to explain the ATP effects on reduction of the parasite index infection shown here (Fig 1) and elsewhere [29], since the activation of P2X7 receptors in macrophages is involved with K^+^ efflux via mechanisms involving connexin/pannexin hemichannels [38]. However, this mechanism does not apply for the P2Y receptor response since P2Y_2_, P2Y_4_ or P2Y_6_ activation does not activate connexin, pannexin hemichannels nor exocytosis in macrophages.

Based on infection index data obtained using selective agonists and antagonists of different subtypes of P2Y family, the P2Y_2_, P2Y_4_ and P2Y_6_ could be considered candidates for mediating uracyl nucleotide effects in macrophages during *T*. *gondii* infection.

In conclusion, UTP and UDP treatments induced tachyzoite egress from macrophages, in a Ca^2+^-dependent manner, and egressed parasites failed to develop novel macrophage infections. Probably because of the activation of P2Y receptors led to incapacity of parasites to actively invade the host cell. This step is associated with a cascade of effects that leads to parasite destruction. Also, P2Y activation in infected cells interfered with parasite cell cycle progression, blocking the replication of the parasites that remained inside host cells. Thus, our data point out for the relevance of pyrimidinergic signaling contribution for innate immune system response against infection and include the P2Y receptors as a new target for development of drugs against *T*. *gondii* infection.

## Supporting Information

S1 Fig Nucleotide treatment has control effect in *T*. *gondii* infected macrophage from different mice lineage. Mouse peritoneal macrophages from BALB/c, C57BL/6 or Swiss Webster were infected with *T*. *gondii* tachyzoites for 2h and then treated with nucleotides for 30 minutes. Treatment with UTP reduced the percentage of infected cells and the number of parasites per host cell (infection index); in a dose-dependent manner. The effect was observed in all mice strain tested (A, B and C). Data represent standard error of mean (SEM) of five independent experiments. * p < 0.05; * * p < 0.001; * * * p < 0.0001.
(TIF)

S1 Video Parasite actively egress from UTP treated cell culture. Peritoneal macrophages were infected with *T*. *gondii* tachyzoites for 2h and then treated with 100 μM UTP. Cell culture was recorded immediately after nucleotide treatment. Video microscopy shows at least 3 parasites egressing from different infected cells.
(MPG)
