# Supplementary figures and images for: Pyrimidinergic Receptor Activation Controls Toxoplasma gondii Infection in Macrophages
Source: PLoS One. 2015 Jul 20;10(7):e0133502. doi: 10.1371/journal.pone.0133502 (PMC4507979; doi:10.1371/journal.pone.0133502)

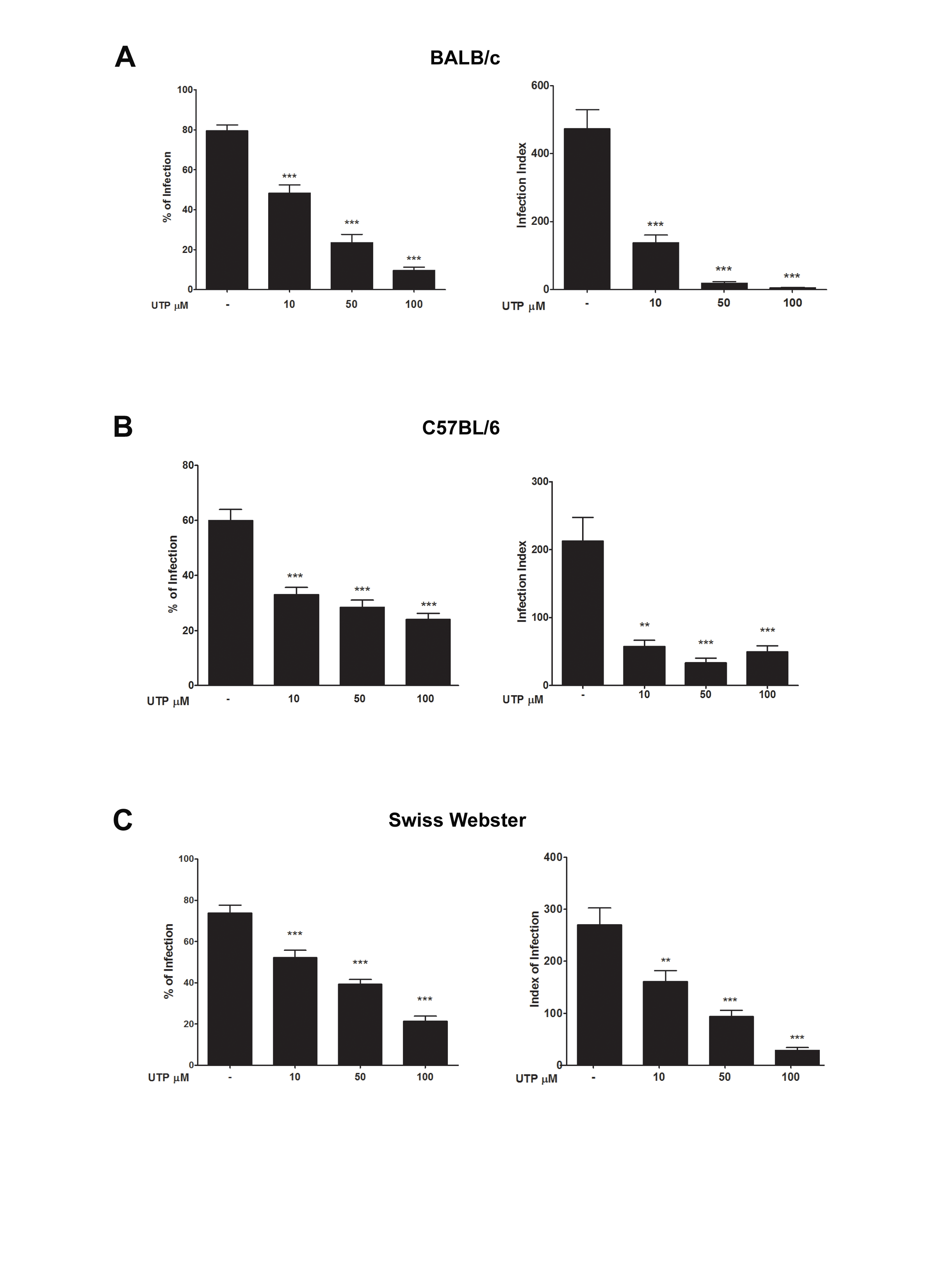

Supplement: S1 Fig — Mouse peritoneal macrophages from BALB/c, C57BL/6 or Swiss Webster were infected with T. gondii tachyzoites for 2h and then treated with nucleotides for 30 minutes. Treatment with UTP reduced the percentage of infected cells and the number of parasites per host cell (infection index); in a dose-dependent manner. The effect was observed in all mice strain tested (A, B and C). Data represent standard error of mean (SEM) of five independent experiments. * p < 0.05; * * p < 0.001; * * * p < 0.0001. (TIF) [file pone.0133502.s001.tif]
